# Selection and evaluation of reference genes for RT-qPCR normalization across developmental stages and tissues in *Dermestes maculatus* (Coleoptera: Dermestidae)

**DOI:** 10.1093/jisesa/ieag114

**Published:** 2026-10-15

**Authors:** Lele Tian, Yang Xia, Guojing Xu, Yinghao Zhai, Yanjie Shang, Yadong Guo

**Affiliations:** Department of Forensic Science, Xiangya School of Basic Medical Sciences, Central South University, Changsha, Hunan, China; Department of Forensic Science, Xiangya School of Basic Medical Sciences, Central South University, Changsha, Hunan, China; Department of Forensic Science, Xiangya School of Basic Medical Sciences, Central South University, Changsha, Hunan, China; Department of Forensic Science, Xiangya School of Basic Medical Sciences, Central South University, Changsha, Hunan, China; Department of Forensic Science, Xiangya School of Basic Medical Sciences, Central South University, Changsha, Hunan, China; Department of Forensic Science, Xiangya School of Basic Medical Sciences, Central South University, Changsha, Hunan, China

**Keywords:** housekeeping genes, expression stability evaluation, stored-product pests, forensic entomology, normalization

## Abstract

Real-time quantitative polymerase chain reaction (RT-qPCR) is widely used for gene expression quantification, and stably expressed reference genes are essential for accurate data normalization. However, validated reference genes are not yet available for the hide beetle, *Dermestes maculatus* De Geer, 1774 (Coleoptera: Dermestidae), an economically important stored-product pest and a forensically relevant necrophagous beetle. In this study, 8 candidate reference genes (*RPL4*, *RPL13A*, *RPL27*, *RPS3*, *RPS18*, *RPS28b*, *GAPDH*, and *EF1α*) were systematically evaluated across 8 developmental stages, 6 tissue types, and all samples. All primer pairs showed good amplification efficiency and specificity. Expression stability was assessed using the comparative ΔCt method, BestKeeper, NormFinder, and geNorm, and the rankings were integrated using RefFinder. The resulting rankings showed that *RPS28b* was the most stable gene across developmental stages, whereas *RPL4* ranked first across tissues and all samples. Pairwise variation analysis using geNorm showed that 2 reference genes were sufficient for normalization in all 3 datasets, supporting *RPL4* and *RPS28b* as the optimal reference-gene combination. *GAPDH* was consistently the least stable gene in all datasets. Validation of *PMP7* expression supported *RPL4* and *RPS28b* as suitable reference genes, whereas *GAPDH* altered the apparent expression pattern. This study provides the first validated RT-qPCR normalization framework for *D. maculatus* and supports future studies of developmental regulation, RNA interference, functional genomics, pest management, and forensic entomology.

## Introduction

The hide beetle, *Dermestes maculatus* De Geer, 1774 (Coleoptera: Dermestidae), is a globally distributed species of economic and forensic importance. Both larvae and adults feed on dry, protein-rich animal-derived materials, including dried meat, leather, animal specimens, and animal or human remains (Schroeder et al. 2002, von Hoermann et al. 2011). Owing to its strong adaptability and reproductive capacity, *D. maculatus* is an important pest of stored animal products and natural history collections, making studies of its biology and control strategies economically valuable (Bellemare and Brunelle 1950, Rajendran and Hajira Parveen 2005). In addition, *D. maculatus* commonly occurs from the late decomposition stage through skeletonization and is therefore a representative necrophagous beetle of forensic importance (Charabidze et al. 2014, Shao et al. 2024). In recent years, laboratory-rearing methods and developmental data under different temperatures have been reported for this species (Richardson and Goff 2001, Zanetti et al. 2016, Martín-Vega et al. 2017, Sanger Ciarleglio et al. 2020, Hu et al. 2024), and it has been used as entomological evidence to estimate the minimum postmortem interval (PMImin) in actual death investigations (Wang et al. 2019, Wang et al. 2021, Pereira et al. 2025). *Dermestes maculatus* has also been proposed as an intermediate-germ beetle model for evolutionary developmental biology, and experimental systems for embryological observation and RNA interference (RNAi) have been established, highlighting its potential for comparative developmental biology and gene-function studies (Xiang et al. 2015, Xiang et al. 2016). Despite its broad research value, molecular studies of *D. maculatus* remain limited, and standardized tools for quantitative gene-expression analysis are still lacking.

Real-time quantitative polymerase chain reaction (RT-qPCR) is one of the most widely used techniques for quantitative gene-expression analysis because of its high sensitivity, strong specificity, low sample requirement, and good reproducibility (Kubista et al. 2006). In insect molecular biology, RT-qPCR has been widely applied to studies of development, metamorphosis, digestive metabolism, detoxification, immunity, RNAi, and environmental adaptation (Lü et al. 2018). It is also frequently used to validate transcriptomic results and to compare target-gene expression patterns among developmental stages, tissues, and experimental treatments (Shakeel et al. 2018). However, the accuracy of RT-qPCR data can be affected by RNA quality, reverse-transcription efficiency, template input, PCR amplification efficiency, and biological variation among samples (Valasek and Repa 2005, Udvardi et al. 2008). More importantly, the selection and validation of stably expressed reference genes are essential for obtaining reliable gene-expression data. Accurate and reproducible RT-qPCR analysis therefore requires appropriate experimental design, validated primer performance, and reliable reference-gene normalization (Vandesompele et al. 2002, Bustin et al. 2009).

Housekeeping genes (HKGs) involved in basic cellular functions are commonly used as reference genes in RT-qPCR, including *actin*, glyceraldehyde-3-phosphate dehydrogenase (*GAPDH*), elongation factor 1 alpha (*EF1α*), ribosomal protein genes, and tubulin genes (Thellin et al. 1999, Lü et al. 2018). However, increasing evidence indicates that no universal reference gene is suitable for all species, tissue types, developmental stages, or experimental conditions (Volkov et al. 2003, Guénin et al. 2009). Even classical HKGs may vary substantially under particular biological conditions. Their use without prior validation may lead to overestimation or underestimation of target-gene ­expression or even produce misleading expression patterns (Vandesompele et al. 2002). Reference-gene validation has been conducted in several coleopteran insects, including *Tribolium castaneum* (Herbst, 1797) (Coleoptera: Tenebrionidae) (Toutges et al. 2010, Ma et al. 2026), *Leptinotarsa decemlineata* (Say, 1824) (Coleoptera: Chrysomelidae) (Shi et al. 2013), *Harmonia axyridis* (Pallas, 1773) (Coleoptera: Coccinellidae) (Yang et al. 2018), and *Epicauta gorhami* (Lewis, 1879) (Coleoptera: Meloidae) (Yang et al. 2024). These studies consistently show that optimal reference genes are species- and condition-dependent and may vary among tissue types, developmental stages, and experimental treatments. Traditional reference genes such as *actin* and *EF1α* are therefore not necessarily stable under all conditions. Systematic screening of reference genes under specific biological conditions has consequently become an essential prerequisite for RT-qPCR analysis in insects. However, such studies remain scarce in Dermestidae, and no RT-qPCR reference genes have yet been systematically validated in *D. maculatus*, limiting the standardization, accuracy, and comparability of subsequent gene-expression studies in this species.

Therefore, this study evaluated 8 candidate reference genes, including ribosomal protein L4 (*RPL4*), ribosomal protein L13A (*RPL13A*), ribosomal protein L27 (*RPL27*), ribosomal protein S3 (*RPS3*), ribosomal protein S18 (*RPS18*), ribosomal protein S28b (*RPS28b*), *GAPDH*, and *EF1α*, across different developmental stages and tissues of *D. maculatus*. Candidate-gene stability was assessed using the comparative ΔCt method, BestKeeper, geNorm, NormFinder, and RefFinder, and suitable reference-gene combinations were identified for different sample types. The results provide validated normalization strategies for future RT-qPCR analyses in *D. maculatus*. To the best of our knowledge, this study represents the first systematic selection and evaluation of RT-qPCR reference genes in a dermestid beetle. The validated reference genes provide a reliable basis for future gene-expression studies in *D. maculatus*, including research on developmental biology, forensic entomology, and pest management.

## Materials and Methods

### Insect rearing

*Dermestes maculatus* was collected in 2024 from Ningxiang, Hunan Province, China (27°55′–28°29′ N, 111°53′–112°46′ E). The collected beetles were transported to the laboratory and used to establish a colony. The colony was maintained in a controlled-environment chamber at 25.0 ± 0.5 °C, 50 ± 10% relative humidity, and a 12:12 h light:dark photoperiod. Air-dried pork slices were provided as food.

### Developmental stage and tissue sampling

To evaluate the stability of candidate reference genes across developmental stages, samples were collected from first- through fifth-instar larvae (L1–L5), wandering larvae (W), pupae (P), and adults (A), representing 8 developmental stages. Three independent biological replicates were prepared for each stage, with each replicate consisting of 10 individuals from the same developmental stage. Individuals were randomly sampled without regard to sex.

Tissue samples were collected from fifth-instar larvae and adults reared for 10 d after emergence. Individuals were rinsed with nuclease-free water, immersed in 75% ethanol for 3 min, and then transferred to chilled phosphate-buffered saline (PBS) for dissection. Six tissue categories were collected: larval head (LH), larval remaining body (LB), larval gut (LG), adult head (AH), adult remaining body (AB), and adult gut (AG). Three independent biological replicates were prepared for each tissue category, with each replicate consisting of pooled tissues from 10 individuals. Individuals were randomly sampled without regard to sex. All samples were immediately snap-frozen in liquid nitrogen and stored at −80 °C until RNA extraction.

### RNA extraction and cDNA synthesis

Total RNA was extracted using the SteadyPure Quick RNA Extraction Kit (catalog no. AG21023; Accurate Biotechnology (Hunan) Co., Ltd., Changsha, China) according to the manufacturer’s instructions. Genomic DNA was removed using the gDNA Clean component of the Evo M-MLV RT Mix Kit with gDNA Clean for qPCR Ver. 2 (catalog no. AG11728; Accurate Biotechnology) at 42 °C for 2 min according to the manufacturer’s instructions. After genomic DNA removal, RNA concentration and purity were measured using a NanoDrop One spectrophotometer (Thermo Fisher Scientific, Waltham, MA, USA). For each sample, 1 μg of total RNA was used for reverse transcription at 37 °C for 15 min followed by 85 °C for 5 s. The synthesized cDNA was diluted tenfold with RNase-free water and stored at −20 °C until use.

### Candidate reference gene identification and primer design

Based on previous reference-gene studies in *T. castaneum* and other coleopteran insects, 8 candidate reference genes were selected: *RPL4*, *RPL13A*, *RPL27*, *RPS3*, *RPS18*, *RPS28b*, *GAPDH*, and *EF1α* (Arakane et al. 2009, Lord et al. 2010, Yang et al. 2024). The corresponding protein sequences of *T. castaneum* were retrieved from the National Center for Biotechnology Information (NCBI) database and used as queries in BLASTp searches against an in-house protein database generated from the annotated genome of *D. maculatus*. Candidate homologs were identified based on sequence similarity and functional annotation. The corresponding cDNA sequences were subsequently retrieved from an in-house *D. maculatus* transcriptome database and used for primer design. Gene-specific primers were designed using Primer Premier 5.0 and synthesized by Accurate Biotechnology (Hunan) Co., Ltd. The cDNA sequences of the 8 candidate reference genes were deposited in GenBank under accession numbers PZ666275–PZ666282. Primer sequences, amplicon sizes, and GenBank accession numbers are provided in Table 1.

**Table 1.** Primer information and amplification characteristics of 8 candidate reference genes in *D. maculatus*

Gene	Gene description	Primer sequences (5′ to 3′)	Product length (bp)	Efficiency (%)	*R*²	Accession no.
***RPL4***	Ribosomal protein L4	F: AACCCAAGATGGCCAACACT R: AGGATTGAGGCGACGTACAC	113	105.66	0.9984	PZ666278
***RPL13A***	Ribosomal protein L13A	F: GAAGGCGTACCACCACCATA R: CGTGACAGTACTTGCGTCCT	97	109.42	0.9993	PZ666277
***RPL27***	Ribosomal protein L27	F: TTGGTCCTAGGTGGCCGATA R: TCAATTCCAGCAACGATGGC	107	108.99	0.9990	PZ666276
***RPS3***	Ribosomal protein S3	F: AAACGGCCACTTCCAGACAA R: GGTTTGATGTCGCTGCTTGG	83	109.69	0.9985	PZ666280
***RPS18***	Ribosomal protein S18	F: GAAGAAGATCCGTGCCCACA R: CGTCTTCCGGTTGTCTTGGT	84	105.18	0.9962	PZ666281
***RPS28b***	Ribosomal protein S28b	F: CCCAGGGACAATGTACGCAA R: TGGCTTCACGTTCAGATTCCA	129	104.21	0.9940	PZ666279
***GAPDH***	Glyceraldehyde-3-phosphate dehydrogenase	F: GCGAGTTAGGTGGTGGTGAT R: TGCTTTCATTCGTCTGGTGGT	133	104.24	0.9945	PZ666282
***EF1α***	Elongation factor 1 alpha	F: GATGCTGCCAATCCAATCCG R: ACAGCACTGTAACCAGCACA	95	102.86	0.9950	PZ666275

### RT-qPCR and primer efficiency

RT-qPCR was performed using the SYBR Green Premix Pro Taq HS qPCR Kit (ROX Plus) (catalog no. AG11718; Accurate Biotechnology) on an ABI 7500 Real-Time PCR System (Applied Biosystems, USA). Each 20-μl reaction contained 10 μl of 2× SYBR Green Pro Taq HS Premix, 0.4 μl of forward primer (10 μM), 0.4 μl of reverse primer (10 μM), 2 μl of tenfold-diluted cDNA, and 7.2 μl of RNase-free water. The amplification program consisted of an initial denaturation at 95 °C for 30 s, followed by 40 cycles of 95 °C for 5 s and 60 °C for 30 s. Melting-curve analysis was subsequently performed in continuous mode at 95 °C for 15 s and 60 °C for 1 min, followed by a ramp from 60 to 95 °C at 1% of the maximum ramp rate with a 30-s hold at 95 °C, and a final step at 60 °C for 15 s. A no-template control (NTC), in which cDNA was replaced with RNase-free water, was included for each primer pair.

Primer amplification efficiency and specificity were evaluated using a 5-point, 5-fold serial dilution of pooled cDNA (1, 1:5, 1:25, 1:125, and 1:625). Standard curves were generated by plotting Ct values against the log10-transformed relative template concentration, and amplification efficiency and the coefficient of determination (*R*²) were calculated (Pfaffl 2001). For *RPL4*, primer amplification efficiency was additionally evaluated at an annealing/extension temperature of 62 °C. Amplification specificity was assessed based on the presence of a single specific peak in the melting curve. All RT-qPCR assays included 3 biological replicates, each with 3 technical replicates. Raw Ct values for all biological samples and technical replicates are provided in Supplementary Table S1.

### Reference gene stability analysis

The expression stability of the candidate reference genes was evaluated in 3 datasets. The developmental-stage dataset comprised 8 developmental stages and 24 independent biological samples. The tissue dataset comprised 6 tissue categories and 18 independent biological samples. The all-sample dataset comprised all 42 biological samples.

Reference-gene stability was evaluated using the comparative ΔCt method, BestKeeper, NormFinder, and geNorm. The comparative ΔCt method evaluates expression stability by calculating the standard deviation of Ct differences between each candidate gene and the other candidate genes. A lower mean standard deviation indicates greater expression stability (Silver et al. 2006). BestKeeper calculates the standard deviation and coefficient of variation from raw Ct values, with a lower standard deviation generally indicating more stable expression (Pfaffl et al. 2004). NormFinder calculates a stability value based on intra- and intergroup variation, with lower values indicating greater stability (Andersen et al. 2004). The geNorm algorithm calculates the average expression ­stability value (*M*) and ranks the candidate genes by sequentially excluding the gene with the highest *M* value. A lower *M* value indicates greater expression stability (Vandesompele et al. 2002).

The rankings generated by the 4 methods were integrated using RefFinder. RefFinder calculates the geometric mean of the ranks obtained from the individual methods, with a lower geometric mean indicating greater overall expression stability (Xie et al. 2023). In addition, geNorm calculates pairwise variation values (*Vn*/*n* + 1) to evaluate the effect of adding another reference gene to the normalization factor. A value of 0.15 was used as the reference threshold for determining the recommended number of reference genes (Vandesompele et al. 2002). When *Vn*/*n* + 1 was less than 0.15, inclusion of the (*n* + 1)th reference gene was considered to provide limited improvement in normalization. Ct-value distributions, stability rankings, RefFinder comprehensive rankings, and geNorm pairwise variation results were visualized using OriginPro 2024.

### Validation of reference genes

To validate the selected reference genes, the expression of a peritrophic matrix protein gene (*PMP7*), previously identified as a gut-enriched gene in our in-house transcriptomic data, was examined in larval and adult tissues. The primer sequences were 5′-ACGGATCTGCATCTCCAACTC-3′ (forward) and 5′-GCATTCGCCGCAATATCCTG-3′ (reverse). *PMP7* expression was examined in larval head (LH), remaining body (LB), and gut (LG), and adult head (AH), remaining body (AB), and gut (AG), with 3 independent biological replicates per tissue and 3 technical replicates per biological replicate. Expression was normalized using either the recommended reference-gene combination (*RPL4* and *RPS28b*) or the least stable candidate, *GAPDH*. For the 2-reference-gene normalization, the mean Ct value of *RPL4* and *RPS28b* was used. Relative expression was calculated using the 2^−ΔΔCt^ method, with the mean ΔCt values of LH and AH used as calibrators for larval and adult tissue analyses, respectively. Statistical comparisons were performed on ΔCt values using 2-sided Welch’s *t*-tests, and *P* values were adjusted using the Holm method for multiple comparisons.

## Results

### Primer specificity and amplification efficiency

Melting-curve analysis of all 8 primer pairs produced a single, distinct peak, with no evident nonspecific amplification peaks, indicating good amplification specificity (Supplementary Fig. S1). Standard curves were generated using a 5-point, 5-fold serial dilution. Ct values for all candidate genes showed strong linear relationships with the logarithm of template concentration (Supplementary Fig. S2). Amplicon sizes ranged from 83 to 133 bp, amplification efficiencies ranged from 102.86% to 109.69%, and *R*² ranged from 0.9940 to 0.9993 (Table 1). These results supported the use of all 8 primer pairs in subsequent reference-gene stability analyses.

### Expression profiles of 8 candidate reference genes

All 8 candidate reference genes were detected across the developmental-stage and tissue samples, but their expression levels and variation differed among genes (Fig. 1). The 6 ribosomal protein genes showed relatively low Ct values, with mean Ct values ranging from 17.83 to 19.31 across developmental stages and from 18.56 to 19.89 across tissues. Among them, *RPL27* showed the lowest mean Ct values, with mean Ct values of 17.83 and 18.56 in the developmental-stage and tissue datasets, respectively. *EF1α* exhibited moderately higher Ct values (22.44 and 22.80, respectively). Among the candidate genes, *RPL4* exhibited the lowest variation across developmental stages (SD = 0.38), while *RPS28b* showed the lowest variation across tissues (SD = 0.42). In contrast, *GAPDH* displayed pronounced variation among developmental stages and tissues. Its mean Ct value was approximately 29.17 to 30.80 from L1 to L5, but decreased to 21.74 in wandering larvae, 19.71 in pupae, and 20.54 in adults. Across tissues, mean *GAPDH* Ct values ranged from 18.88 in the adult gut to 31.27 in the larval gut. Accordingly, *GAPDH* showed the greatest overall Ct variation in both the developmental-stage dataset (SD = 4.55) and the tissue dataset (SD = 4.57). These results indicated substantial differences in transcript abundance and expression variability among the candidate genes, supporting further stability assessment using multiple algorithms.

**Fig. 1.**
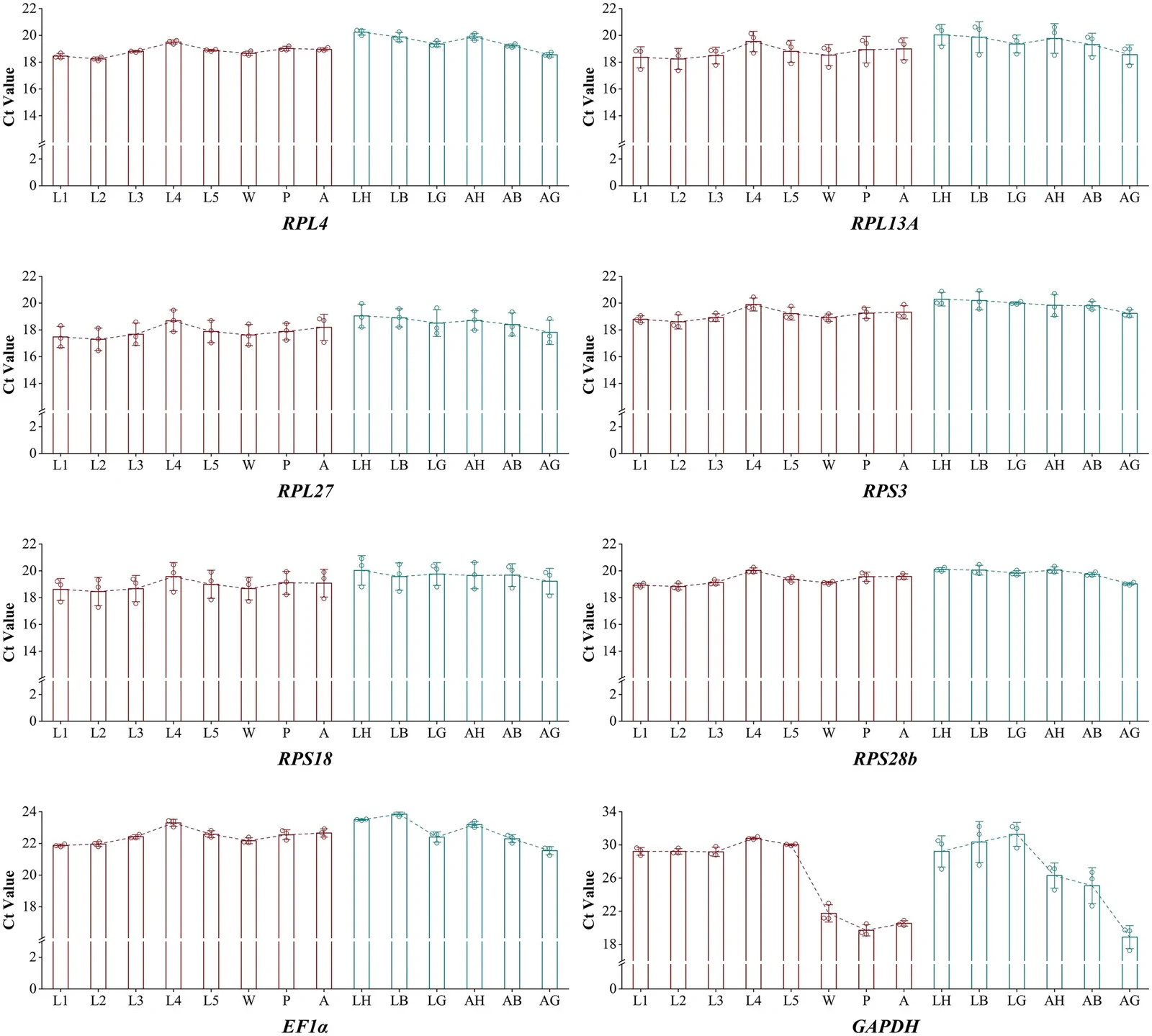
Ct values of 8 candidate reference genes across developmental stages and tissues of *D. maculatus*. Bars represent the mean ± SD of 3 biological replicates, and each point represents an individual biological replicate. L1–L5, first- to fifth-instar larvae; W, wandering larvae; P, pupae; A, adults; LH, larval head; LB, larval remaining body; LG, larval gut; AH, adult head; AB, adult remaining body; AG, adult gut.

### Stability analysis and comprehensive ranking of candidate reference genes

Across developmental stages, the 4 algorithms produced partially consistent stability rankings. The comparative ΔCt method ranked *RPS28b* and *EF1α* as the 2 most stable genes. BestKeeper ranked *RPL4* and *RPS28b* highest, whereas NormFinder ranked *EF1α* and *RPS3* as the top 2 genes. The geNorm analysis jointly ranked *RPS28b* and *RPL4* as the most stable pair. In contrast, *GAPDH* was consistently ranked as the least stable gene by all 4 algorithms (Table 2). RefFinder identified *RPS28b* as the most stable gene, followed by *RPL4*, *EF1α*, *RPS3*, *RPL13A*, *RPS18*, *RPL27*, and *GAPDH* (Fig. 2A).

**Fig. 2.**
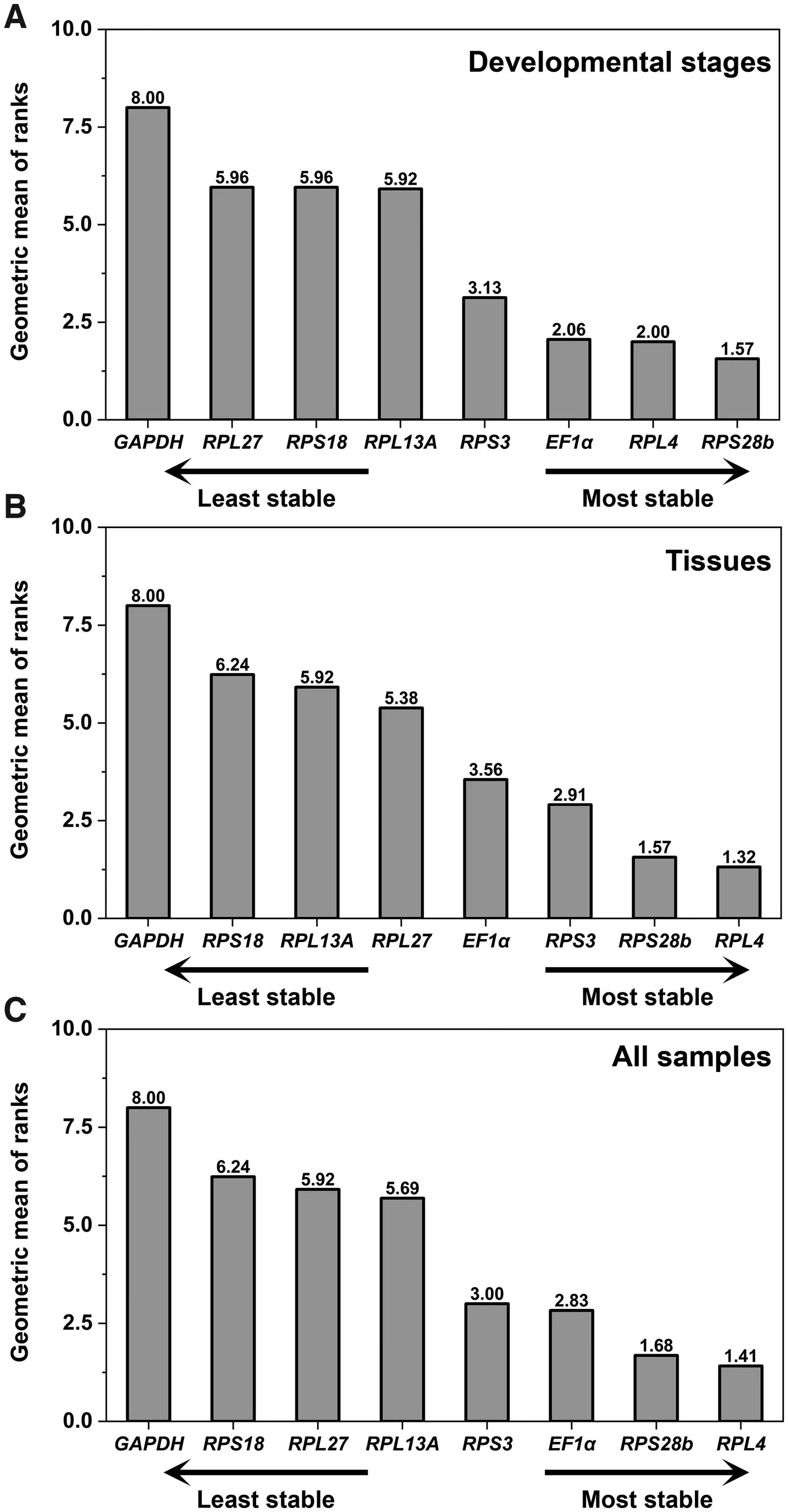
Comprehensive stability ranking of 8 candidate reference genes in *D. maculatus* using RefFinder. A) Developmental-stage samples; B) tissue samples; C) all samples. The comprehensive ranking was determined from the geometric mean of the ranks generated by the comparative ΔCt method, BestKeeper, NormFinder, and geNorm. Lower geometric mean values indicate greater expression stability. Candidate genes are arranged from the least stable to the most stable.

**Table 2.** Expression stability values and rankings of 8 candidate reference genes in *D. maculatus* across developmental-stage samples, tissue samples, and all samples

Conditions	Genes	ΔCt		BestKeeper		NormFinder		geNorm	
		Stability	Rank	Stability	Rank	Stability	Rank	Stability	Rank
**Developmental stages**	*RPS28b*	1.037	1	0.421	2	0.623	3	0.163	1
	*RPL4*	1.048	4	0.384	1	0.634	4	0.163	1
	*EF1α*	1.040	2	0.466	3	0.564	1	0.209	3
	*RPS3*	1.047	3	0.517	4	0.605	2	0.266	4
	*RPL13A*	1.343	7	0.783	5	1.167	5	0.797	7
	*RPL27*	1.334	6	0.799	6	1.177	7	0.711	5
	*RPS18*	1.322	5	0.879	7	1.171	6	0.685	6
	*GAPDH*	4.619	8	4.545	8	3.996	8	4.619	8
**Tissues**	*RPL4*	1.090	1	0.603	3	0.479	1	0.255	1
	*RPS28b*	1.097	2	0.422	1	0.564	3	0.255	1
	*RPS3*	1.133	3	0.561	2	0.571	4	0.416	3
	*EF1α*	1.222	4	0.834	5	0.540	2	0.574	4
	*RPL27*	1.410	6	0.823	4	1.223	7	0.810	5
	*RPL13A*	1.365	5	0.907	7	0.907	5	0.925	7
	*RPS18*	1.413	7	0.849	6	1.218	6	0.820	6
	*GAPDH*	4.246	8	4.567	8	3.183	8	4.246	8
**All samples**	*RPL4*	1.070	1	0.599	2	0.574	2	0.232	1
	*RPS28b*	1.074	2	0.483	1	0.589	4	0.232	1
	*EF1α*	1.149	4	0.665	4	0.537	1	0.442	4
	*RPS3*	1.092	3	0.658	3	0.588	3	0.345	3
	*RPL13A*	1.345	5	0.909	6	1.057	5	0.850	7
	*RPL27*	1.358	7	0.878	5	1.199	7	0.757	5
	*RPS18*	1.354	6	0.936	7	1.186	6	0.746	6
	*GAPDH*	4.411	8	4.507	8	3.661	8	4.411	8

In the tissue dataset, the comparative ΔCt method ranked *RPL4* and *RPS28b* highest, BestKeeper ranked *RPS28b* and *RPS3* highest, whereas NormFinder ranked *RPL4* and *EF1α* as the top 2 genes. The geNorm analysis jointly identified *RPL4* and *RPS28b* as the most stable pair. *GAPDH* ranked last in all analyses (Table 2). RefFinder ranked the genes as follows: *RPL4*, *RPS28b*, *RPS3*, *EF1α*, *RPL27*, *RPL13A*, *RPS18*, and *GAPDH* (Fig. 2B).

In the all-sample dataset, the comparative ΔCt method ranked *RPL4* and *RPS28b* as the 2 most stable genes. BestKeeper also ranked *RPS28b* and *RPL4* highest, whereas NormFinder ranked *EF1α* and *RPL4* as the top 2 genes. The geNorm analysis jointly ranked *RPL4* and *RPS28b* as the most stable pair. *GAPDH* was again consistently identified as the least stable gene by all 4 algorithms (Table 2). RefFinder ranked *RPL4* as the most stable gene, followed by *RPS28b*, *EF1α*, *RPS3*, *RPL13A*, *RPL27*, *RPS18*, and *GAPDH* (Fig. 2C).

### Optimal number of reference genes

Pairwise variation analysis showed that 2 reference genes were sufficient for stable normalization in all 3 datasets. In the developmental-stage dataset, V2/3 was 0.064, below the 0.15 threshold, indicating that 2 reference genes were sufficient. In the tissue dataset, V2/3 was 0.133, also below 0.15. Similarly, in the all-sample dataset, V2/3 was 0.109, supporting the use of 2 reference genes (Fig. 3; Supplementary Table S2). Based on the geNorm pairwise variation results and RefFinder rankings, *RPS28b* and *RPL4* were recommended for developmental-stage samples, whereas *RPL4* and *RPS28b* were recommended for both tissue samples and all samples. Thus, the same 2-gene combination was supported across all 3 datasets.

**Fig. 3.**
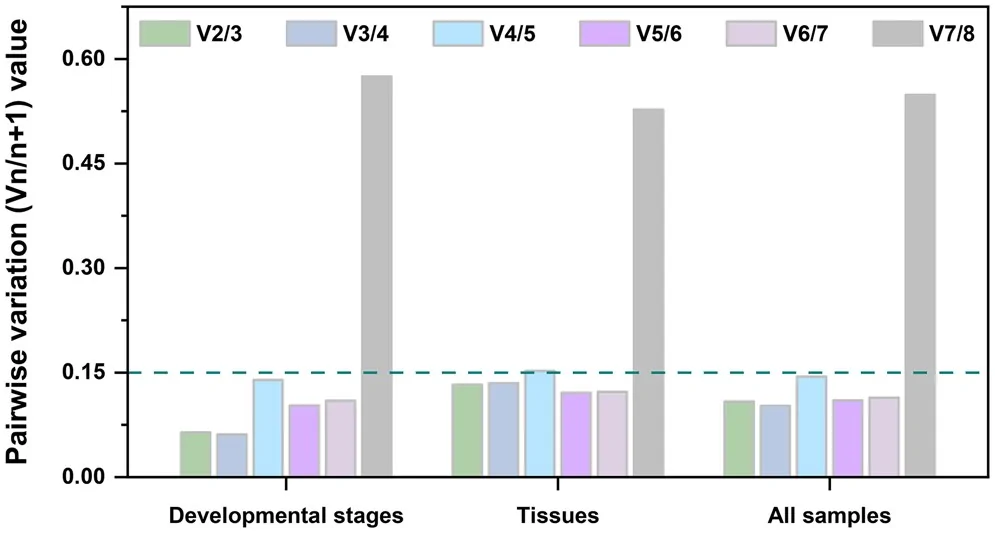
Pairwise variation analysis using geNorm to determine the optimal number of reference genes in *D. maculatus*. Values of *Vn*/*n* + 1 are shown for developmental stages, tissues, and all samples. The dashed line indicates the 0.15 threshold. Values below 0.15 indicate that inclusion of an additional reference gene provides limited improvement in the normalization factor.

### Validation of selected reference genes

Normalization with *RPL4* and *RPS28b* revealed clear gut-enriched expression of *PMP7* in both larvae and adults (Fig. 4). In larvae, *PMP7* expression was significantly higher in the gut than in the head (Holm-adjusted P = 0.006) and remaining body (P = 0.006). Similarly, in adults, expression in the gut was significantly higher than that in the head (P < 0.001) and remaining body (P < 0.001). In contrast, normalization with *GAPDH* altered the apparent expression pattern. Although larval gut expression remained significantly higher than that in the head (P = 0.011) and remaining body (P = 0.035), the adult gut showed lower apparent expression than the head and remaining body, with neither comparison reaching statistical significance (P = 0.099 and P = 0.164, respectively). These results further support the suitability of *RPL4* and *RPS28b* for RT-qPCR normalization in *D. maculatus* tissues.

**Fig. 4.**
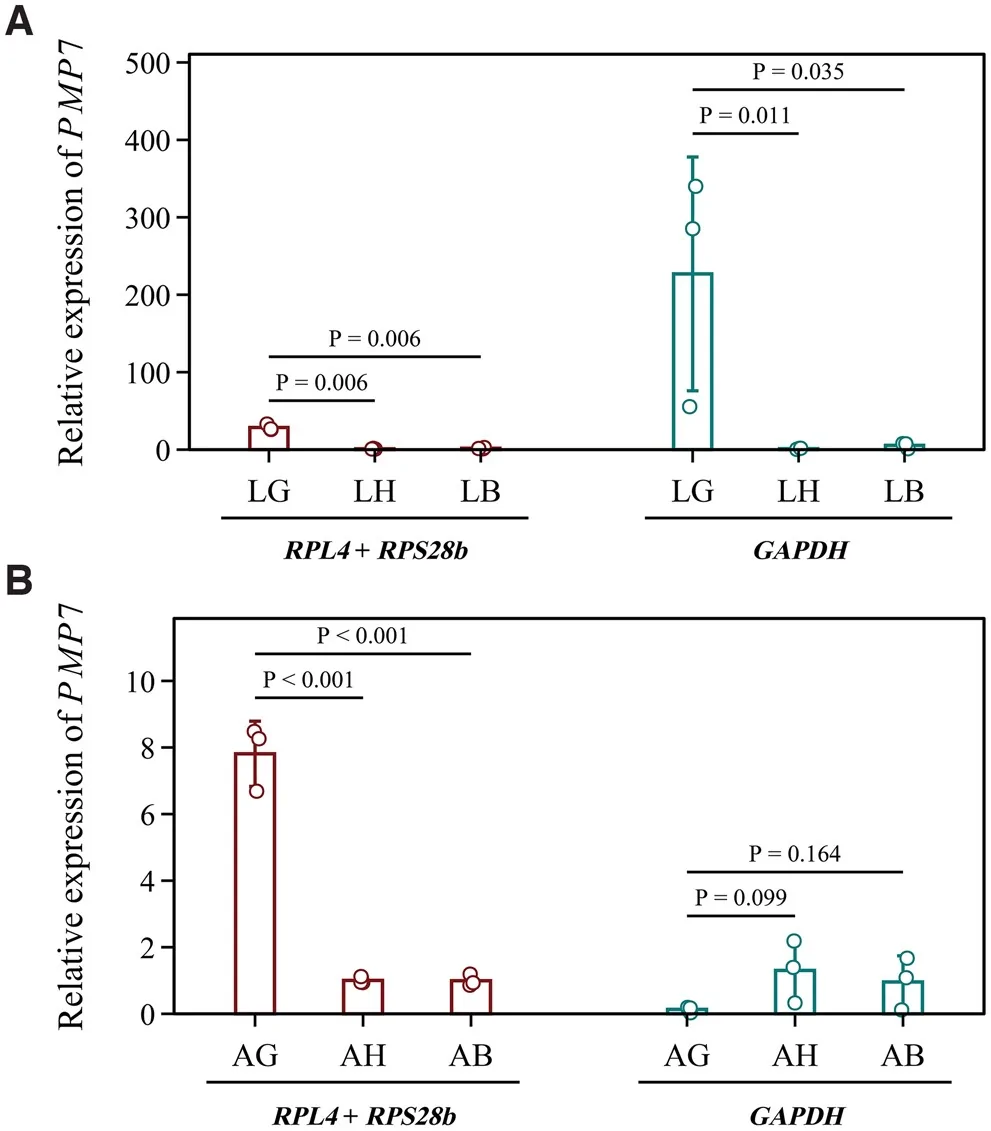
Validation of reference genes using *PMP7* expression in larval (A) and adult (B) tissues of *D. maculatus*. Expression was normalized using *RPL4* + *RPS28b* or *GAPDH*. Bars show mean ± SD of 3 biological replicates, and each point represents an individual biological replicate. *P* values were obtained from 2-sided Welch’s *t*-tests on ΔCt values and adjusted using the Holm method. LG, larval gut; LH, larval head; LB, larval remaining body; AG, adult gut; AH, adult head; AB, adult remaining body.

## Discussion

This study systematically evaluated the expression stability of 8 candidate reference genes in *D. maculatus* across developmental stages, tissue types, and all samples. Although the individual algorithms showed some differences in gene ranking, the integrated RefFinder analysis consistently identified *RPL4* and *RPS28b* among the most stable candidates. *RPS28b* ranked first in the developmental-stage dataset, whereas *RPL4* ranked first in both the tissue and all-sample datasets. Moreover, geNorm pairwise variation analysis indicated that 2 reference genes were sufficient for normalization in all 3 datasets, supporting the combination of *RPL4* and *RPS28b*. In contrast, *GAPDH* ranked last in all 3 datasets. These findings support *RPL4* and *RPS28b* as a suitable reference-gene combination and highlight the need for reference-gene validation in RT-qPCR studies.

The most stable candidate genes identified in this study were mainly members of the ribosomal protein gene family. Ribosomal proteins are essential components of ribosomes and participate in ribosome assembly, maturation, and protein translation (Ferreira-Cerca et al. 2005). Previous studies have similarly shown that some ribosomal protein genes exhibit relatively stable expression under specific biological conditions and can serve as suitable RT-qPCR reference genes. For example, *RPS3*, *RPS18*, and *RPL13A* were identified as relatively stable reference genes in *T. castaneum* following fungal challenge, whereas *β-actin*, *α-tubulin*, and ribosomal protein S6 (*RPS6*) showed lower stability (Lord et al. 2010). In *L. decemlineata*, the ribosomal protein genes *RP4* and *RP18* also showed high stability (Shi et al. 2013). In *Mylabris sibirica* (Fischer von Waldheim, 1823) (Coleoptera: Meloidae), ribosomal protein L6 (*RPL6*) and ribosomal protein L13 (*RPL13*) were the most stable reference genes overall across adult ages, adult tissues, temperatures, and sexes (Shen et al. 2024). These findings suggest that ribosomal protein genes may have considerable potential for normalization in coleopteran insects.

Notably, *GAPDH* showed substantial Ct-value variation in the developmental-stage, tissue, and all-sample datasets and was consistently ranked as the least stable candidate by all algorithms. This result is consistent with findings in other coleopteran species. For example, all algorithms identified *GAPDH* as the least stable gene in *Oxycetonia jucunda* (Faldermann, 1835) (Coleoptera: Scarabaeidae) (Zhao et al. 2026). *GAPDH* is a highly conserved gene encoding a key glycolytic enzyme, and its expression may be influenced by differences in tissue energy metabolism, developmental status, and physiological activity (Barber et al. 2005). *Dermestes maculatus* undergoes complete metamorphosis, and the head, gut, and remaining body also differ substantially in cellular composition and metabolic function. These differences may partly explain the marked variation in *GAPDH* expression. In addition, *GAPDH* expression has been reported to exhibit circadian rhythmicity, and under alternating light–dark conditions, sampling time may therefore contribute to its expression variability (Shinohara et al. 1998, Figueredo et al. 2018). However, this finding does not imply that *GAPDH* is unsuitable as a reference gene in all insects or under all experimental conditions. For example, *GAPDH* was recommended as a reference gene under different temperature treatments in *H. axyridis* (Yang et al. 2018). Overall, in *D. maculatus*, *GAPDH* should not be used as a single reference gene for developmental-stage or tissue-expression analyses without prior validation.

The geNorm pairwise variation analysis estimated the number of reference genes required for stable normalization in each dataset. The use of multiple validated reference genes and the geometric mean of their expression levels can reduce normalization bias caused by variation in a single reference gene (Vandesompele et al. 2002). In this study, V2/3 values were below 0.15 in the developmental-stage (0.064), tissue (0.133), and all-sample (0.109) datasets, indicating that 2 reference genes were sufficient for normalization across all 3 datasets. Together with the RefFinder rankings, these results support the use of *RPL4* and *RPS28b* in combination under the conditions evaluated here.

The validated reference-gene framework established in this study provides a basic tool for molecular research in *D. maculatus* and may support studies of stored-product pest management and forensic entomology. Embryonic and parental RNAi methods have already been established in this species, and RNAi has been used to investigate the expression and functions of segmentation genes (Xiang et al. 2015). The reference genes validated here can therefore be used to assess RNAi knockdown efficiency and support quantitative gene-expression analyses. Nevertheless, the present study evaluated candidate reference genes only across developmental stages and tissues. Their stability should be further assessed under additional conditions, including different temperatures and insecticide exposure, to establish reliable normalization strategies for broader applications. Overall, this study systematically selected and validated RT-qPCR reference-gene combinations for developmental-stage and tissue analyses in *D. maculatus*, providing a reliable methodological basis for future research on developmental regulation, tissue-specific expression, RNA interference, functional genomics, pest management, and forensic entomology.

## Supplementary Material

ieag114_Supplementary_Data

## Data Availability

The data supporting the findings of this study are available within the article and its online supplementary material. The nucleotide sequences of the 8 candidate reference genes have been deposited in the GenBank Nucleotide Database under accession numbers PZ666275–PZ666282. Additional raw RT-qPCR data are available from the corresponding author upon reasonable request.
