# Supplementary material for: Selection and evaluation of reference genes for RT-qPCR normalization across developmental stages and tissues in Dermestes maculatus (Coleoptera: Dermestidae)
Source: J Insect Sci. 2026 Oct 15;26(5):ieag114. doi: 10.1093/jisesa/ieag114 (PMC13668882; doi:10.1093/jisesa/ieag114)
Supplement: Supplementary file 1 [file ieag114_supplementary_data.docx]

**Selection and evaluation of reference genes for RT-qPCR normalization across developmental stages and tissues in *Dermestes maculatus* (Coleoptera: Dermestidae)**

**Supplementary Material**

**Fig. S1.** Melting curves of eight candidate reference genes in *Dermestes maculatus*. Each primer pair produced a single distinct peak, indicating specific amplification of *RPL4*, *RPL13A*, *RPL27*, *RPS3*, *RPS18*, *RPS28b*, *GAPDH*, and *EF1α*.

**Alt text:** Eight melting-curve plots, one for each candidate reference gene, show a single distinct peak for every primer pair, indicating specific amplification without evident secondary peaks.

**Fig. S2.** Standard curves for eight candidate reference genes in *Dermestes maculatus*. Ct values were plotted against the log10-transformed relative cDNA concentration. The regression equation and coefficient of determination (R²) are shown for each candidate gene.

**Alt text:** Eight standard-curve plots show Ct values decreasing linearly with increasing log-transformed cDNA concentration. All genes display strong linear relationships, with R² values ranging from 0.9940 to 0.9993.

**Table S1.** Raw Ct values of eight candidate reference genes across developmental stage and tissue samples of *Dermestes maculatus*.

| **Sample ID** | **Group description** | **Gene** | **Technical replicate 1** | **Technical replicate 2** | **Technical replicate 3** |
| --- | --- | --- | --- | --- | --- |
| L1_Bio1 | First-instar larva | *RPL4* | 18.65962410 | 18.64167786 | 18.72045517 |
| L1_Bio1 | First-instar larva | *RPL13A* | 18.93884468 | 18.76081848 | 18.69779396 |
| L1_Bio1 | First-instar larva | *RPL27* | 17.30395889 | 17.37641907 | 17.45988655 |
| L1_Bio1 | First-instar larva | *RPS3* | 18.99534035 | 19.05307198 | 19.14027405 |
| L1_Bio1 | First-instar larva | *RPS18* | 19.30902672 | 19.21944046 | 19.08105850 |
| L1_Bio1 | First-instar larva | *RPS28b* | 19.30858612 | 18.74830627 | 19.14700508 |
| L1_Bio1 | First-instar larva | *GAPDH* | 29.39491844 | 29.92645645 | 29.60113335 |
| L1_Bio1 | First-instar larva | *EF1α* | 22.23811150 | 22.02415657 | 21.64925575 |
| L1_Bio2 | First-instar larva | *RPL4* | 18.35926628 | 18.29284286 | 18.46300888 |
| L1_Bio2 | First-instar larva | *RPL13A* | 17.42182541 | 17.43151474 | 17.50354004 |
| L1_Bio2 | First-instar larva | *RPL27* | 18.51222229 | 18.18760872 | 18.22372818 |
| L1_Bio2 | First-instar larva | *RPS3* | 18.43537140 | 18.53130722 | 18.68944359 |
| L1_Bio2 | First-instar larva | *RPS18* | 19.01344681 | 18.92353821 | 18.91956329 |
| L1_Bio2 | First-instar larva | *RPS28b* | 18.90371704 | 18.79536438 | 18.64700508 |
| L1_Bio2 | First-instar larva | *GAPDH* | 28.81032753 | 28.63978004 | 28.71007729 |
| L1_Bio2 | First-instar larva | *EF1α* | 21.83830261 | 21.90871429 | 21.82642937 |
| L1_Bio3 | First-instar larva | *RPL4* | 18.23373795 | 18.25491142 | 18.46485901 |
| L1_Bio3 | First-instar larva | *RPL13A* | 18.83566666 | 18.91378784 | 18.76961327 |
| L1_Bio3 | First-instar larva | *RPL27* | 16.74807739 | 16.70256042 | 16.75059891 |
| L1_Bio3 | First-instar larva | *RPS3* | 18.76842880 | 18.74537277 | 18.79071426 |
| L1_Bio3 | First-instar larva | *RPS18* | 17.75245667 | 17.62236786 | 17.64384079 |
| L1_Bio3 | First-instar larva | *RPS28b* | 18.97365952 | 18.87043571 | 18.94636345 |
| L1_Bio3 | First-instar larva | *GAPDH* | 29.12959099 | 29.45010757 | 29.38305092 |
| L1_Bio3 | First-instar larva | *EF1α* | 21.81739044 | 21.77847481 | 21.77498245 |
| L2_Bio1 | Second-instar larva | *RPL4* | 18.33919144 | 18.35763931 | 18.45219231 |
| L2_Bio1 | Second-instar larva | *RPL13A* | 18.92300606 | 18.79206085 | 18.93685341 |
| L2_Bio1 | Second-instar larva | *RPL27* | 17.30590630 | 17.23254776 | 17.41049194 |
| L2_Bio1 | Second-instar larva | *RPS3* | 19.18350410 | 19.20635033 | 19.25111008 |
| L2_Bio1 | Second-instar larva | *RPS18* | 19.46587563 | 19.16558838 | 19.29681396 |
| L2_Bio1 | Second-instar larva | *RPS28b* | 19.14628029 | 18.99942017 | 19.13548279 |
| L2_Bio1 | Second-instar larva | *GAPDH* | 29.11555099 | 29.03230858 | 28.93846130 |
| L2_Bio1 | Second-instar larva | *EF1α* | 22.10159302 | 21.99678230 | 22.20366669 |
| L2_Bio2 | Second-instar larva | *RPL4* | 18.17337418 | 18.31088066 | 18.22447777 |
| L2_Bio2 | Second-instar larva | *RPL13A* | 17.33556175 | 17.35349846 | 17.40392685 |
| L2_Bio2 | Second-instar larva | *RPL27* | 18.07539558 | 18.09189606 | 18.18334579 |
| L2_Bio2 | Second-instar larva | *RPS3* | 18.29499054 | 18.32844353 | 18.41152382 |
| L2_Bio2 | Second-instar larva | *RPS18* | 18.7979126 | 18.68938255 | 18.85838127 |
| L2_Bio2 | Second-instar larva | *RPS28b* | 18.73878098 | 18.41453362 | 18.72304153 |
| L2_Bio2 | Second-instar larva | *GAPDH* | 29.02146721 | 29.07003212 | 29.00585365 |
| L2_Bio2 | Second-instar larva | *EF1α* | 21.95533371 | 21.97027588 | 22.06202888 |
| L2_Bio3 | Second-instar larva | *RPL4* | 18.07509041 | 18.02356339 | 18.19104004 |
| L2_Bio3 | Second-instar larva | *RPL13A* | 18.41024399 | 18.38407135 | 18.63182259 |
| L2_Bio3 | Second-instar larva | *RPL27* | 16.42082596 | 16.35351944 | 16.56421852 |
| L2_Bio3 | Second-instar larva | *RPS3* | 18.18387032 | 18.23421478 | 18.31636047 |
| L2_Bio3 | Second-instar larva | *RPS18* | 17.19750023 | 17.31592751 | 17.29187012 |
| L2_Bio3 | Second-instar larva | *RPS28b* | 18.74342346 | 18.66997337 | 18.84879875 |
| L2_Bio3 | Second-instar larva | *GAPDH* | 29.53642845 | 29.41600418 | 29.87447929 |
| L2_Bio3 | Second-instar larva | *EF1α* | 21.71829033 | 21.97485733 | 21.69761086 |
| L3_Bio1 | Third-instar larva | *RPL4* | 18.81590080 | 18.87697792 | 18.92894936 |
| L3_Bio1 | Third-instar larva | *RPL13A* | 18.77392769 | 18.93881798 | 18.91230392 |
| L3_Bio1 | Third-instar larva | *RPL27* | 17.44327927 | 17.48875427 | 17.53151894 |
| L3_Bio1 | Third-instar larva | *RPS3* | 19.17811775 | 19.34907532 | 19.22921562 |
| L3_Bio1 | Third-instar larva | *RPS18* | 19.28941345 | 19.49570465 | 19.34399414 |
| L3_Bio1 | Third-instar larva | *RPS28b* | 19.16073418 | 19.43283463 | 19.43752861 |
| L3_Bio1 | Third-instar larva | *GAPDH* | 28.75026131 | 28.76379776 | 28.84480667 |
| L3_Bio1 | Third-instar larva | *EF1α* | 22.39722443 | 22.65419960 | 22.65862846 |
| L3_Bio2 | Third-instar larva | *RPL4* | 18.77700615 | 18.72316742 | 18.94035530 |
| L3_Bio2 | Third-instar larva | *RPL13A* | 17.67435455 | 17.86201286 | 17.77493477 |
| L3_Bio2 | Third-instar larva | *RPL27* | 18.58699036 | 18.58629799 | 18.59430122 |
| L3_Bio2 | Third-instar larva | *RPS3* | 18.68563461 | 18.78890610 | 18.80013657 |
| L3_Bio2 | Third-instar larva | *RPS18* | 19.11171341 | 19.14579964 | 18.98839378 |
| L3_Bio2 | Third-instar larva | *RPS28b* | 18.96655655 | 19.10328865 | 19.01709366 |
| L3_Bio2 | Third-instar larva | *GAPDH* | 29.09829521 | 28.85559654 | 28.82209396 |
| L3_Bio2 | Third-instar larva | *EF1α* | 22.30064201 | 22.33310127 | 22.40869713 |
| L3_Bio3 | Third-instar larva | *RPL4* | 18.70678902 | 18.46021271 | 19.03430939 |
| L3_Bio3 | Third-instar larva | *RPL13A* | 18.53839111 | 18.95964241 | 18.98526192 |
| L3_Bio3 | Third-instar larva | *RPL27* | 16.84349632 | 16.95024490 | 17.06263733 |
| L3_Bio3 | Third-instar larva | *RPS3* | 18.70452309 | 18.71691895 | 18.83603096 |
| L3_Bio3 | Third-instar larva | *RPS18* | 17.41271019 | 17.56754684 | 17.67133141 |
| L3_Bio3 | Third-instar larva | *RPS28b* | 18.52889252 | 19.27318001 | 19.19560242 |
| L3_Bio3 | Third-instar larva | *GAPDH* | 29.77770233 | 29.78647041 | 29.85889244 |
| L3_Bio3 | Third-instar larva | *EF1α* | 22.21348381 | 22.39307785 | 22.44271088 |
| L4_Bio1 | Fourth-instar larva | *RPL4* | 19.48664093 | 19.53882599 | 19.62350845 |
| L4_Bio1 | Fourth-instar larva | *RPL13A* | 20.06266594 | 20.16890144 | 20.15565491 |
| L4_Bio1 | Fourth-instar larva | *RPL27* | 18.55301476 | 18.71261597 | 18.83694077 |
| L4_Bio1 | Fourth-instar larva | *RPS3* | 20.39957809 | 20.43532753 | 20.50744438 |
| L4_Bio1 | Fourth-instar larva | *RPS18* | 20.49506187 | 20.49726486 | 20.34876823 |
| L4_Bio1 | Fourth-instar larva | *RPS28b* | 20.09059715 | 20.30230713 | 20.39162636 |
| L4_Bio1 | Fourth-instar larva | *GAPDH* | 31.20516777 | 30.76725769 |  |
| L4_Bio1 | Fourth-instar larva | *EF1α* | 23.31791115 | 23.43229103 | 23.48169708 |
| L4_Bio2 | Fourth-instar larva | *RPL4* | 19.19635201 | 19.37286377 | 19.44289589 |
| L4_Bio2 | Fourth-instar larva | *RPL13A* | 18.63151360 | 18.62784195 | 18.77744484 |
| L4_Bio2 | Fourth-instar larva | *RPL27* | 19.36992455 | 19.54879761 | 19.48117638 |
| L4_Bio2 | Fourth-instar larva | *RPS3* | 19.50448608 | 19.58975601 | 19.58987808 |
| L4_Bio2 | Fourth-instar larva | *RPS18* | 19.76337433 | 19.88286591 | 19.94160843 |
| L4_Bio2 | Fourth-instar larva | *RPS28b* | 19.73981285 | 19.98117447 | 20.05688095 |
| L4_Bio2 | Fourth-instar larva | *GAPDH* | 30.70791054 | 30.92184639 | 30.62575531 |
| L4_Bio2 | Fourth-instar larva | *EF1α* | 23.44675827 | 23.41801643 | 23.46019554 |
| L4_Bio3 | Fourth-instar larva | *RPL4* | 19.95859528 | 19.34014893 | 19.56177711 |
| L4_Bio3 | Fourth-instar larva | *RPL13A* | 19.45960617 | 19.96076393 | 20.03695107 |
| L4_Bio3 | Fourth-instar larva | *RPL27* | 17.76872063 | 17.86927032 | 17.92442322 |
| L4_Bio3 | Fourth-instar larva | *RPS3* | 19.55376053 | 19.70185852 | 19.77506065 |
| L4_Bio3 | Fourth-instar larva | *RPS18* | 18.40669250 | 18.38449860 | 18.44300842 |
| L4_Bio3 | Fourth-instar larva | *RPS28b* | 19.79682159 | 19.90653038 | 19.98295403 |
| L4_Bio3 | Fourth-instar larva | *GAPDH* | 30.43677521 | 30.63102150 | 30.88746262 |
| L4_Bio3 | Fourth-instar larva | *EF1α* | 22.92030334 | 23.08463097 | 23.11691475 |
| L5_Bio1 | Fifth-instar larva | *RPL4* | 18.95191574 | 18.94667053 | 18.92913246 |
| L5_Bio1 | Fifth-instar larva | *RPL13A* | 19.41355896 | 19.43177795 | 19.42003441 |
| L5_Bio1 | Fifth-instar larva | *RPL27* | 17.85029984 | 17.98959732 | 17.95319176 |
| L5_Bio1 | Fifth-instar larva | *RPS3* | 19.73888016 | 19.77217865 | 19.80676460 |
| L5_Bio1 | Fifth-instar larva | *RPS18* | 19.82592010 | 19.93361855 | 19.92287636 |
| L5_Bio1 | Fifth-instar larva | *RPS28b* | 19.44048500 | 19.60611343 | 19.59900284 |
| L5_Bio1 | Fifth-instar larva | *GAPDH* | 29.89340210 | 30.06870079 | 30.36549759 |
| L5_Bio1 | Fifth-instar larva | *EF1α* | 22.82172585 | 22.78540039 | 22.85612869 |
| L5_Bio2 | Fifth-instar larva | *RPL4* | 18.75838661 | 18.79039574 | 18.85376930 |
| L5_Bio2 | Fifth-instar larva | *RPL13A* | 17.82776642 | 17.90345764 | 17.89662933 |
| L5_Bio2 | Fifth-instar larva | *RPL27* | 18.68143082 | 18.68854713 | 18.67378998 |
| L5_Bio2 | Fifth-instar larva | *RPS3* | 18.95072174 | 18.92049026 | 18.90940285 |
| L5_Bio2 | Fifth-instar larva | *RPS18* | 19.16034698 | 19.35403061 | 19.22565651 |
| L5_Bio2 | Fifth-instar larva | *RPS28b* | 19.03510857 | 19.17861938 | 19.21298027 |
| L5_Bio2 | Fifth-instar larva | *GAPDH* | 30.12473297 | 30.01837158 | 30.03322411 |
| L5_Bio2 | Fifth-instar larva | *EF1α* | 22.52215004 | 22.61428833 | 22.47003365 |
| L5_Bio3 | Fifth-instar larva | *RPL4* | 19.48724556 | 18.59641838 | 18.65382004 |
| L5_Bio3 | Fifth-instar larva | *RPL13A* | 19.14530182 | 19.11820030 | 19.08561897 |
| L5_Bio3 | Fifth-instar larva | *RPL27* | 17.00944138 | 17.06662178 | 16.99740982 |
| L5_Bio3 | Fifth-instar larva | *RPS3* | 18.95432663 | 18.94567680 | 18.93752861 |
| L5_Bio3 | Fifth-instar larva | *RPS18* | 17.81816101 | 17.87227821 | 17.81052017 |
| L5_Bio3 | Fifth-instar larva | *RPS28b* | 19.41585732 | 19.48804092 | 19.29364204 |
| L5_Bio3 | Fifth-instar larva | *GAPDH* | 29.79755592 | 29.91115189 | 29.95387840 |
| L5_Bio3 | Fifth-instar larva | *EF1α* | 22.50351524 | 22.30145264 | 22.36593628 |
| W_Bio1 | Wandering larva | *RPL4* | 18.69718170 | 18.87825966 | 18.94161224 |
| W_Bio1 | Wandering larva | *RPL13A* | 18.96905327 | 18.95928192 | 18.84511948 |
| W_Bio1 | Wandering larva | *RPL27* | 17.41371727 | 17.49040985 | 17.74413490 |
| W_Bio1 | Wandering larva | *RPS3* | 19.23941803 | 19.11813164 | 19.23093033 |
| W_Bio1 | Wandering larva | *RPS18* | 19.24987793 | 19.45318794 | 19.30298996 |
| W_Bio1 | Wandering larva | *RPS28b* | 19.17879486 | 19.28334618 | 19.18491173 |
| W_Bio1 | Wandering larva | *GAPDH* | 20.98241425 | 21.25514984 |  |
| W_Bio1 | Wandering larva | *EF1α* | 22.39505196 | 22.39872742 | 22.37917519 |
| W_Bio2 | Wandering larva | *RPL4* | 18.47520638 | 18.60103226 | 18.78316498 |
| W_Bio2 | Wandering larva | *RPL13A* | 17.66714859 | 17.61676979 | 17.53468704 |
| W_Bio2 | Wandering larva | *RPL27* | 18.33103561 | 18.43792343 | 18.54459000 |
| W_Bio2 | Wandering larva | *RPS3* | 18.54789734 | 18.66972160 | 18.72022820 |
| W_Bio2 | Wandering larva | *RPS18* | 18.97393227 | 18.99066925 | 18.95932198 |
| W_Bio2 | Wandering larva | *RPS28b* | 18.98635101 | 19.03286171 | 18.97316170 |
| W_Bio2 | Wandering larva | *GAPDH* | 21.05663490 | 21.22112465 | 21.21019554 |
| W_Bio2 | Wandering larva | *EF1α* | 22.11400604 | 22.14224815 | 22.02472496 |
| W_Bio3 | Wandering larva | *RPL4* | 18.38594055 | 18.55326271 | 18.60202980 |
| W_Bio3 | Wandering larva | *RPL13A* | 19.01089287 | 19.08027077 | 19.03837013 |
| W_Bio3 | Wandering larva | *RPL27* | 16.72306061 | 16.80006027 | 17.06529999 |
| W_Bio3 | Wandering larva | *RPS3* | 18.82617760 | 18.83976173 | 18.97069550 |
| W_Bio3 | Wandering larva | *RPS18* | 17.78049088 | 17.65005493 | 17.72078133 |
| W_Bio3 | Wandering larva | *RPS28b* | 19.06272507 | 19.19688606 | 19.07773018 |
| W_Bio3 | Wandering larva | *GAPDH* | 22.93775749 | 22.96685982 | 22.93433952 |
| W_Bio3 | Wandering larva | *EF1α* | 21.94154930 | 22.06459808 | 22.09741974 |
| P_Bio1 | Pupa | *RPL4* | 19.07123184 | 19.21901512 | 19.33629799 |
| P_Bio1 | Pupa | *RPL13A* | 19.37566948 | 19.43830872 | 19.38319016 |
| P_Bio1 | Pupa | *RPL27* | 17.85359001 | 17.97220230 | 17.99188805 |
| P_Bio1 | Pupa | *RPS3* | 19.52049828 | 19.65950012 | 19.68621635 |
| P_Bio1 | Pupa | *RPS18* | 19.95292473 | 19.91404152 | 19.92321968 |
| P_Bio1 | Pupa | *RPS28b* | 19.83681679 | 19.96080399 | 19.73045158 |
| P_Bio1 | Pupa | *GAPDH* | 19.37773705 | 19.38710022 | 19.48563576 |
| P_Bio1 | Pupa | *EF1α* | 22.92836571 | 22.74773598 | 22.74684143 |
| P_Bio2 | Pupa | *RPL4* | 18.86421776 | 18.88022423 | 18.95121002 |
| P_Bio2 | Pupa | *RPL13A* | 17.88701057 | 17.79264832 | 17.71714020 |
| P_Bio2 | Pupa | *RPL27* | 18.31462479 | 18.54776764 | 18.53613091 |
| P_Bio2 | Pupa | *RPS3* | 18.75054359 | 18.84010315 | 18.85075569 |
| P_Bio2 | Pupa | *RPS18* | 19.28328896 | 19.11598015 | 19.0831604 |
| P_Bio2 | Pupa | *RPS28b* | 19.25466347 | 19.21527481 | 19.05249786 |
| P_Bio2 | Pupa | *GAPDH* | 19.16461372 | 19.21520424 | 19.29371643 |
| P_Bio2 | Pupa | *EF1α* | 22.73740196 | 22.65816689 | 22.51238632 |
| P_Bio3 | Pupa | *RPL4* | 18.97415543 | 18.89004326 | 18.96021461 |
| P_Bio3 | Pupa | *RPL13A* | 19.59356308 | 19.59106064 | 19.66632652 |
| P_Bio3 | Pupa | *RPL27* | 17.21349907 | 17.22438622 | 17.22644806 |
| P_Bio3 | Pupa | *RPS3* | 19.20178986 | 19.26320457 | 19.48114777 |
| P_Bio3 | Pupa | *RPS18* | 18.12067604 | 18.35099983 | 18.18977928 |
| P_Bio3 | Pupa | *RPS28b* | 19.71841240 | 19.64998817 | 19.62465096 |
| P_Bio3 | Pupa | *GAPDH* | 20.47103119 | 20.47729111 | 20.50084686 |
| P_Bio3 | Pupa | *EF1α* | 22.17066574 | 22.26422691 | 22.17068672 |
| A_Bio1 | Adult | *RPL4* | 19.02809143 | 19.05142975 | 19.16546822 |
| A_Bio1 | Adult | *RPL13A* | 19.63316917 | 19.47898293 | 19.66110802 |
| A_Bio1 | Adult | *RPL27* | 18.77066803 | 18.81023026 | 18.87858963 |
| A_Bio1 | Adult | *RPS3* | 19.83117294 | 19.88422394 | 19.96170044 |
| A_Bio1 | Adult | *RPS18* | 19.89751053 | 19.84552193 | 19.94750786 |
| A_Bio1 | Adult | *RPS28b* | 19.88936996 | 19.68968773 | 19.84632111 |
| A_Bio1 | Adult | *GAPDH* | 20.18844604 | 20.37207794 |  |
| A_Bio1 | Adult | *EF1α* | 22.85151482 | 22.93692780 | 22.96059990 |
| A_Bio2 | Adult | *RPL4* | 18.93181419 | 18.86903572 | 18.93647385 |
| A_Bio2 | Adult | *RPL13A* | 17.99818993 | 18.05738831 | 18.11690903 |
| A_Bio2 | Adult | *RPL27* | 18.67530251 | 18.66484451 | 18.73508072 |
| A_Bio2 | Adult | *RPS3* | 19.02190018 | 18.99618530 | 19.10279083 |
| A_Bio2 | Adult | *RPS18* | 19.48640442 | 19.30308533 | 19.48695564 |
| A_Bio2 | Adult | *RPS28b* | 19.57377815 | 19.33700371 | 19.50425148 |
| A_Bio2 | Adult | *GAPDH* | 20.39554214 | 20.48901176 | 20.40625572 |
| A_Bio2 | Adult | *EF1α* | 22.68139458 | 22.59669304 | 22.73019600 |
| A_Bio3 | Adult | *RPL4* | 18.94842720 | 18.79481888 | 18.85526085 |
| A_Bio3 | Adult | *RPL13A* | 19.28922081 | 19.33828163 | 19.36896706 |
| A_Bio3 | Adult | *RPL27* | 16.94332314 | 17.10003662 | 17.12829590 |
| A_Bio3 | Adult | *RPS3* | 18.99254799 | 18.97683716 | 19.10356712 |
| A_Bio3 | Adult | *RPS18* | 17.91399574 | 17.92428589 | 17.89452171 |
| A_Bio3 | Adult | *RPS28b* | 19.47927666 | 19.36823082 | 19.52695084 |
| A_Bio3 | Adult | *GAPDH* | 20.88197899 | 20.87910843 | 20.94668007 |
| A_Bio3 | Adult | *EF1α* | 22.37141037 | 22.44302940 | 22.37965012 |
| LH_Bio1 | Larval head | *RPL4* | 20.21836090 | 20.30183029 | 20.55204964 |
| LH_Bio1 | Larval head | *RPL13A* | 20.53136635 | 20.56870270 | 20.70810127 |
| LH_Bio1 | Larval head | *RPL27* | 18.86875916 | 18.94621277 | 19.00766182 |
| LH_Bio1 | Larval head | *RPS3* | 20.89878654 | 20.80984879 | 20.92408371 |
| LH_Bio1 | Larval head | *RPS18* | 20.88239288 | 20.86299706 | 20.96995163 |
| LH_Bio1 | Larval head | *RPS28b* | 20.19094658 | 20.20576859 | 20.37040710 |
| LH_Bio1 | Larval head | *GAPDH* | 30.48913002 | 30.80743027 | 30.21146965 |
| LH_Bio1 | Larval head | *EF1α* | 23.43109512 | 23.49162674 | 23.42026520 |
| LH_Bio2 | Larval head | *RPL4* | 20.41415215 | 20.21057320 | 20.51629066 |
| LH_Bio2 | Larval head | *RPL13A* | 19.03357506 | 19.21152115 | 19.21589661 |
| LH_Bio2 | Larval head | *RPL27* | 19.91467476 | 19.94423676 | 20.04088402 |
| LH_Bio2 | Larval head | *RPS3* | 19.86391258 | 19.99457550 | 20.18456459 |
| LH_Bio2 | Larval head | *RPS18* | 20.51441765 | 20.25374222 | 20.42686272 |
| LH_Bio2 | Larval head | *RPS28b* | 20.00881958 | 20.17717361 | 20.00417709 |
| LH_Bio2 | Larval head | *GAPDH* | 27.07409477 | 27.04704285 | 27.07458305 |
| LH_Bio2 | Larval head | *EF1α* | 23.46494675 | 23.47793007 | 23.67051888 |
| LH_Bio3 | Larval head | *RPL4* | 19.97099495 | 19.89982414 | 20.08260918 |
| LH_Bio3 | Larval head | *RPL13A* | 20.16604996 | 20.43006134 | 20.48837471 |
| LH_Bio3 | Larval head | *RPL27* | 18.08386421 | 18.27468872 | 18.28503227 |
| LH_Bio3 | Larval head | *RPS3* | 19.93480110 | 19.99478912 | 20.02472878 |
| LH_Bio3 | Larval head | *RPS18* | 18.74773788 | 18.76404190 | 18.85397148 |
| LH_Bio3 | Larval head | *RPS28b* | 19.89992905 | 19.98109436 | 20.02201653 |
| LH_Bio3 | Larval head | *GAPDH* | 30.07866287 | 30.09079742 | 30.13177490 |
| LH_Bio3 | Larval head | *EF1α* | 23.39434242 | 23.49079704 | 23.62852287 |
| LB_Bio1 | Larval remaining body | *RPL4* | 20.13007736 | 20.21390533 | 20.44501114 |
| LB_Bio1 | Larval remaining body | *RPL13A* | 20.64727211 | 20.52979088 | 20.69647598 |
| LB_Bio1 | Larval remaining body | *RPL27* | 18.98783493 | 18.84187317 | 18.95507050 |
| LB_Bio1 | Larval remaining body | *RPS3* | 20.87543297 | 20.93916321 | 20.94717026 |
| LB_Bio1 | Larval remaining body | *RPS18* | 20.55384254 | 20.36030006 | 20.52874947 |
| LB_Bio1 | Larval remaining body | *RPS28b* | 20.36842346 | 20.45323944 | 20.58021545 |
| LB_Bio1 | Larval remaining body | *GAPDH* | 32.46094131 | 31.86457253 | 32.33911514 |
| LB_Bio1 | Larval remaining body | *EF1α* | 23.89035606 | 23.97318840 | 23.93156242 |
| LB_Bio2 | Larval remaining body | *RPL4* | 19.49294281 | 19.54194832 | 19.70514107 |
| LB_Bio2 | Larval remaining body | *RPL13A* | 18.40900993 | 18.56973839 | 18.63037682 |
| LB_Bio2 | Larval remaining body | *RPL27* | 19.51286507 | 19.56640816 | 19.62881088 |
| LB_Bio2 | Larval remaining body | *RPS3* | 19.44819260 | 19.54405403 | 19.66816330 |
| LB_Bio2 | Larval remaining body | *RPS18* | 19.69048882 | 19.77988243 | 19.83876991 |
| LB_Bio2 | Larval remaining body | *RPS28b* | 19.52320671 | 19.90091324 | 19.94793320 |
| LB_Bio2 | Larval remaining body | *GAPDH* | 27.50555229 | 27.65672684 | 27.50030327 |
| LB_Bio2 | Larval remaining body | *EF1α* | 23.81293869 | 23.94654846 | 24.01299095 |
| LB_Bio3 | Larval remaining body | *RPL4* | 19.79292107 | 19.78860664 | 19.80236816 |
| LB_Bio3 | Larval remaining body | *RPL13A* | 20.19747353 | 20.82221985 | 20.31369209 |
| LB_Bio3 | Larval remaining body | *RPL27* | 18.15664482 | 18.21072769 | 18.26538467 |
| LB_Bio3 | Larval remaining body | *RPS3* | 20.06910133 | 19.98246956 | 20.24534416 |
| LB_Bio3 | Larval remaining body | *RPS18* | 18.35767365 | 18.47887993 | 18.55157471 |
| LB_Bio3 | Larval remaining body | *RPS28b* | 20.00284386 | 19.46371841 | 20.23023796 |
| LB_Bio3 | Larval remaining body | *GAPDH* | 31.29201508 | 31.25533676 | 31.29999733 |
| LB_Bio3 | Larval remaining body | *EF1α* | 23.44643593 | 23.70285416 | 23.94142532 |
| LG_Bio1 | Larval gut | *RPL4* | 19.12994576 | 19.21826172 | 19.32648849 |
| LG_Bio1 | Larval gut | *RPL13A* | 19.61004639 | 19.48830795 | 19.62323761 |
| LG_Bio1 | Larval gut | *RPL27* | 18.05549240 | 18.15878105 | 18.17332268 |
| LG_Bio1 | Larval gut | *RPS3* | 20.06939888 | 20.15537262 | 20.12877846 |
| LG_Bio1 | Larval gut | *RPS18* | 20.16233826 | 20.10838127 | 20.10807991 |
| LG_Bio1 | Larval gut | *RPS28b* | 19.69012833 | 19.61754990 | 19.70040131 |
| LG_Bio1 | Larval gut | *GAPDH* | 31.70840645 | 32.34184265 | 32.57910538 |
| LG_Bio1 | Larval gut | *EF1α* | 22.53382874 | 22.53944016 | 22.64991951 |
| LG_Bio2 | Larval gut | *RPL4* | 19.49466896 | 19.65454483 | 19.63539314 |
| LG_Bio2 | Larval gut | *RPL13A* | 18.59875107 | 18.59261703 | 18.63383293 |
| LG_Bio2 | Larval gut | *RPL27* | 19.60345078 | 19.73100853 | 19.58835030 |
| LG_Bio2 | Larval gut | *RPS3* | 19.92184830 | 19.99111176 | 19.97451973 |
| LG_Bio2 | Larval gut | *RPS18* | 20.40231705 | 20.31608963 | 20.37265778 |
| LG_Bio2 | Larval gut | *RPS28b* | 19.95474243 | 20.12644005 | 20.04896355 |
| LG_Bio2 | Larval gut | *GAPDH* | 29.84488869 | 29.34566879 | 29.67633438 |
| LG_Bio2 | Larval gut | *EF1α* | 21.98374748 | 21.94973373 | 22.13706779 |
| LG_Bio3 | Larval gut | *RPL4* | 19.25629044 | 19.19892120 | 19.30846977 |
| LG_Bio3 | Larval gut | *RPL13A* | 19.87926292 | 19.87097168 | 19.93301773 |
| LG_Bio3 | Larval gut | *RPL27* | 17.63610077 | 17.76974487 | 17.82983398 |
| LG_Bio3 | Larval gut | *RPS3* | 19.84926605 | 19.90374374 | 19.98990059 |
| LG_Bio3 | Larval gut | *RPS18* | 18.74108315 | 18.75323296 | 18.88166428 |
| LG_Bio3 | Larval gut | *RPS28b* | 19.81230736 | 19.76618385 | 19.75364876 |
| LG_Bio3 | Larval gut | *GAPDH* | 32.42351151 | 31.73486328 | 31.79870605 |
| LG_Bio3 | Larval gut | *EF1α* | 22.60809517 | 22.53425789 | 22.65785980 |
| AH_Bio1 | Adult head | *RPL4* | 20.09906578 | 20.17930984 | 20.14992332 |
| AH_Bio1 | Adult head | *RPL13A* | 20.51761627 | 20.59240723 | 20.69829559 |
| AH_Bio1 | Adult head | *RPL27* | 18.63211823 | 18.80904198 | 18.77884102 |
| AH_Bio1 | Adult head | *RPS3* | 20.77233696 | 20.66705322 | 20.72585106 |
| AH_Bio1 | Adult head | *RPS18* | 20.50015450 | 20.63821220 | 20.71019173 |
| AH_Bio1 | Adult head | *RPS28b* | 20.27849197 | 20.42255592 | 20.32527351 |
| AH_Bio1 | Adult head | *GAPDH* | 27.04726219 | 27.14438057 | 27.20120621 |
| AH_Bio1 | Adult head | *EF1α* | 23.35644341 | 23.26667595 | 23.52530479 |
| AH_Bio2 | Adult head | *RPL4* | 19.70196342 | 19.84178734 | 19.34659767 |
| AH_Bio2 | Adult head | *RPL13A* | 18.41487885 | 18.59687233 | 18.52009773 |
| AH_Bio2 | Adult head | *RPL27* | 19.30394173 | 19.51194382 | 19.42048454 |
| AH_Bio2 | Adult head | *RPS3* | 18.97968674 | 18.95575523 | 19.29763603 |
| AH_Bio2 | Adult head | *RPS18* | 19.6685276 | 19.70095444 | 19.73714447 |
| AH_Bio2 | Adult head | *RPS28b* | 19.84528923 | 20.08397865 | 19.76380920 |
| AH_Bio2 | Adult head | *GAPDH* | 24.47848892 | 24.50551224 | 24.66129875 |
| AH_Bio2 | Adult head | *EF1α* | 23.13272858 | 23.25814247 | 23.15798187 |
| AH_Bio3 | Adult head | *RPL4* | 19.81929016 | 19.85659790 | 20.01213455 |
| AH_Bio3 | Adult head | *RPL13A* | 20.15289116 | 20.23161316 | 20.20625496 |
| AH_Bio3 | Adult head | *RPL27* | 17.95592880 | 17.94090462 | 18.00163460 |
| AH_Bio3 | Adult head | *RPS3* | 19.36173058 | 19.94569397 | 19.81354332 |
| AH_Bio3 | Adult head | *RPS18* | 18.66170120 | 18.56444740 | 18.68954277 |
| AH_Bio3 | Adult head | *RPS28b* | 19.92893600 | 19.98836136 | 19.97124290 |
| AH_Bio3 | Adult head | *GAPDH* | 27.22258759 | 27.21864700 | 27.20809364 |
| AH_Bio3 | Adult head | *EF1α* | 22.84714317 | 23.09176064 | 23.09108162 |
| AB_Bio1 | Adult remaining body | *RPL4* | 19.09644699 | 19.19808006 | 19.25607300 |
| AB_Bio1 | Adult remaining body | *RPL13A* | 19.72282982 | 19.73162270 | 19.75578690 |
| AB_Bio1 | Adult remaining body | *RPL27* | 18.11492538 | 18.39078331 | 18.14833069 |
| AB_Bio1 | Adult remaining body | *RPS3* | 20.10344315 | 20.12853813 | 20.25227356 |
| AB_Bio1 | Adult remaining body | *RPS18* | 20.27923584 | 20.19651794 | 20.42689705 |
| AB_Bio1 | Adult remaining body | *RPS28b* | 19.64818764 | 19.56187630 | 19.74972153 |
| AB_Bio1 | Adult remaining body | *GAPDH* | 26.67875290 | 26.69886589 | 26.71208191 |
| AB_Bio1 | Adult remaining body | *EF1α* | 22.35180283 | 22.67551804 | 22.52308846 |
| AB_Bio2 | Adult remaining body | *RPL4* | 19.33859253 | 19.37530136 | 19.41140938 |
| AB_Bio2 | Adult remaining body | *RPL13A* | 18.20260811 | 18.36795998 | 18.46402931 |
| AB_Bio2 | Adult remaining body | *RPL27* | 19.30301476 | 19.35823441 | 19.36080933 |
| AB_Bio2 | Adult remaining body | *RPS3* | 19.50729179 | 19.50368500 | 19.54082108 |
| AB_Bio2 | Adult remaining body | *RPS18* | 19.97795486 | 19.9661274 | 20.15729904 |
| AB_Bio2 | Adult remaining body | *RPS28b* | 19.98264503 | 19.81288528 | 19.93206024 |
| AB_Bio2 | Adult remaining body | *GAPDH* | 22.53478432 | 22.57933998 | 22.74710846 |
| AB_Bio2 | Adult remaining body | *EF1α* | 21.96779633 | 22.09673119 | 21.97902489 |
| AB_Bio3 | Adult remaining body | *RPL4* | 19.17762756 | 19.16973114 | 18.93630981 |
| AB_Bio3 | Adult remaining body | *RPL13A* | 19.77760315 | 19.86744690 | 19.92183304 |
| AB_Bio3 | Adult remaining body | *RPL27* | 17.67283058 | 17.69676208 | 17.57355499 |
| AB_Bio3 | Adult remaining body | *RPS3* | 19.69929886 | 19.76084709 | 19.78200340 |
| AB_Bio3 | Adult remaining body | *RPS18* | 18.72039986 | 18.69664001 | 18.71818352 |
| AB_Bio3 | Adult remaining body | *RPS28b* | 19.49659920 | 19.67983818 | 19.80382729 |
| AB_Bio3 | Adult remaining body | *GAPDH* | 25.83006477 | 25.98209381 | 25.93172455 |
| AB_Bio3 | Adult remaining body | *EF1α* | 22.30436707 | 22.24363708 | 22.54176140 |
| AG_Bio1 | Adult gut | *RPL4* | 18.43774605 | 18.53766441 | 18.58634758 |
| AG_Bio1 | Adult gut | *RPL13A* | 18.96420860 | 18.98388863 | 18.99123955 |
| AG_Bio1 | Adult gut | *RPL27* | 17.43207932 | 17.63911057 | 17.54285622 |
| AG_Bio1 | Adult gut | *RPS3* | 19.43854141 | 19.59465981 | 19.58112526 |
| AG_Bio1 | Adult gut | *RPS18* | 19.84767342 | 19.94206619 | 19.83350372 |
| AG_Bio1 | Adult gut | *RPS28b* | 18.93065834 | 18.99826050 | 19.15038681 |
| AG_Bio1 | Adult gut | *GAPDH* | 19.58547974 | 19.61737633 | 19.69696045 |
| AG_Bio1 | Adult gut | *EF1α* | 21.51244164 | 21.79404640 | 21.80426407 |
| AG_Bio2 | Adult gut | *RPL4* | 18.66180420 | 18.65617752 | 18.88933754 |
| AG_Bio2 | Adult gut | *RPL13A* | 17.60043526 | 17.79769325 | 17.80336189 |
| AG_Bio2 | Adult gut | *RPL27* | 18.71093559 | 18.83811188 | 18.95612144 |
| AG_Bio2 | Adult gut | *RPS3* | 18.97295761 | 19.05094910 | 19.06552124 |
| AG_Bio2 | Adult gut | *RPS18* | 19.60346031 | 19.67087936 | 19.7472744 |
| AG_Bio2 | Adult gut | *RPS28b* | 19.18626213 | 19.13453484 | 19.08977699 |
| AG_Bio2 | Adult gut | *GAPDH* | 17.22076035 | 17.23317146 | 17.34132004 |
| AG_Bio2 | Adult gut | *EF1α* | 21.16978073 | 21.18372726 | 21.36523056 |
| AG_Bio3 | Adult gut | *RPL4* | 18.58898354 | 18.46515274 | 18.15033340 |
| AG_Bio3 | Adult gut | *RPL13A* | 18.86682129 | 19.08316803 | 18.98503876 |
| AG_Bio3 | Adult gut | *RPL27* | 16.95834541 | 17.31352806 | 16.96069145 |
| AG_Bio3 | Adult gut | *RPS3* | 19.03254890 | 19.14403725 | 19.17447472 |
| AG_Bio3 | Adult gut | *RPS18* | 18.09828568 | 18.11733437 | 18.15059090 |
| AG_Bio3 | Adult gut | *RPS28b* | 18.87992668 | 18.95548439 | 18.93787003 |
| AG_Bio3 | Adult gut | *GAPDH* | 19.72735977 | 19.82696533 | 19.64219666 |
| AG_Bio3 | Adult gut | *EF1α* | 21.63170433 | 21.72620583 |  |

**Note:** Blank cells indicate technical replicates excluded due to excessive variation among technical replicates (SD > 0.5). Mean Ct values were calculated from the remaining valid technical replicates.

**Table S2.** Pairwise variation values for determining the optimal number of reference genes in *D. maculatus*. Pairwise variation values (Vn/n+1) were calculated for developmental-stage samples, tissue samples, and all samples. Values below 0.15 indicate that inclusion of an additional reference gene provides limited improvement in the normalization factor.

| Analysis set | Comparison | Pairwise variation |
| --- | --- | --- |
| All samples | V2/3 | 0.108545419 |
|  | V3/4 | 0.102283587 |
|  | V4/5 | 0.144235985 |
|  | V5/6 | 0.110168432 |
|  | V6/7 | 0.114166711 |
|  | V7/8 | 0.548400465 |
| Developmental stages | V2/3 | 0.064203663 |
|  | V3/4 | 0.061251353 |
|  | V4/5 | 0.139665362 |
|  | V5/6 | 0.10271554 |
|  | V6/7 | 0.109583919 |
|  | V7/8 | 0.574885477 |
| Tissues | V2/3 | 0.132689574 |
|  | V3/4 | 0.134921811 |
|  | V4/5 | 0.15257262 |
|  | V5/6 | 0.120970992 |
|  | V6/7 | 0.122614419 |
|  | V7/8 | 0.527330891 |
